# The Treatment of Fractures from a Common-Sense Point of View

**Published:** 1907-09-21

**Authors:** 


### Points in Surgery.

### THE TREATMENT OF FRACTURES FROM A COMMON-SENSE POINT OF VIEW.

### V. COMPOUND FRACTURES.

There could be no more striking testimony to the value of antiseptic methods in surgery, if any were still needed, than a comparison between the mortality from compound fractures to-day and that of twenty or thirty years ago. In those days it was not uncommon to find that as many as sixty per cent. of the cases of compound fracture terminated fatally; in fact, this injury was regarded with so much dread that many surgeons advocated primary amputation as a routine measure, arguing that it was better that the patient should lose his limb than his life. What happened was that the wound was infected at the time of the accident and the organisms ran riot, attacking not only the soft parts but the ends of the fractured bone, so that the patient ran the risk of losing his life from acute infective osteomyelitis or from pyæmia. The first great principle, therefore, in treating a compound fracture is to render the wound aseptic. For this purpose an anæsthetic should be given in all cases. The skin for a large area round the wound should be well soaped and shaved, washed with ether, and finally scrubbed with a 1-in-20 solution of carbolic acid containing 1 part in 2,000 of biniodide of mercury. The treatment of the wound itself depends largely upon its size. The wound in the skin may be quite small even when a large bone is fractured; both tibia and fibula, for instance, may be involved in a compound fracture while the skin only shows a punctured wound large enough to admit an ordinary probe. In these cases it is most tempting merely to wash the skin in the manner already mentioned, irrigate the wound with antiseptic solution, and then treat the case as an ordinary fracture. It is, of course, true that many cases do very well if treated in this way, but the surgeon who elects to pursue this course undertakes a grave responsibility and one with little to recommend it. If asepsis is not procured (and it is very difficult to be sure that the antiseptic has reached all the contaminated parts through a small aperture) the results are apt to be lamentable indeed. It is a far sounder practice to make an incision along the line of the limb over the site of the fracture of such a length that the condition both of the damaged soft parts and of the fractured ends may be examined, and the whole interior swabbed over with antiseptic fluid. Moreover, if any bleeding is occurring from small vessels which have been ruptured by the violence of the accident, these can be tied, and loose pieces of bone lying between the ends can be removed. The wound should be stitched up again, and, if asepsis has been obtained, it should unite readily. Where a large surface-wound has already been made by the force producing the fracture, a condition commonly met with when the fracture has been caused by direct violence, all foreign matter and dirt must be removed, blood clots carefully sponged away, and the whole wound thoroughly swabbed out with a strong antiseptic solution. If the wound is a contused one the damaged edges should be cut away with a scalpel, to prevent any subsequent sloughing. If any doubt exists as to the vitality of the skin, a simple test is to apply an Esmarch's bandage and tourniquet, which are removed after a minute or two. Healthy skin, which was formerly blanched, becomes suffused, but that in which the vitality is impaired remains white. The fractured ends should then be set in apposition and the limb put up in suitable retentive apparatus. The wound should be sewn up; of course, if much skin has had to be sacrificed, complete apposition of the edges will be impossible, but the raw surface left should be as small as is compatible with the circumstances of the case. If necessary, the deficiency can be remedied later by skin grafting.

There seems to be a superstition that because a fracture is a compound one, the correct procedure is to wire or otherwise mechanically fix the fractured ends. This is far from being the case. The indications for wiring the bone in compound fractures are the same as those already laid down for simple fractures. In any case it is inadvisable to wire the ends of the bone in compound fractures until such time has elapsed that the surgeon can be sure that the wound is aseptic; otherwise necrosis of the bone will certainly occur, and the portions between the wire will separate as a sequestrum, and this will lead to permanent shortening of the limb. If mechanical fixation of the ends is indicated, it is better done after a few days, when the patient has recovered from the shock of the accident and it is certain that the wound is aseptic. Some surgeons advocate the subcutaneous injection of antitetanic serum as a prophylactic measure in all cases in which the wound has been contaminated with earth or dirt from the road. This is a reasonable precaution.

Although primary amputation in compound fractures is no longer regarded as a routine measure, cases occur in which it becomes advisable or necessary. The indications for amputation are regulated by (1) the state of the limb, and (2) the state of the patient. If the limb is partially torn off, it must, of course, be removed, and the stump must be trimmed. If, in addition to extensive comminution of the bones, the main vessels or nerves of the limb have been torn across, it is generally necessary to amputate; but even here it is, perhaps, better to tie the vessels, suture the nerves, and watch the progress of the case. If gangrene occurs, or the limb is quite useless owing to permanent loss of nerve function, amputation can always be performed later. In old and feeble individuals, with sclerosed vessels and defective circulation, conservative attempts expose the patient to a considerable risk of his life by prolonging his stay in bed.

